# Rolling Circle Amplification in Integrated Microsystems: An Uncut Gem toward Massively Multiplexed Pathogen Diagnostics and Genotyping

**DOI:** 10.1021/acs.accounts.1c00438

**Published:** 2021-10-12

**Authors:** Ruben R. G. Soares, Narayanan Madaboosi, Mats Nilsson

**Affiliations:** † Department of Biochemistry and Biophysics, Science for Life Laboratory, Stockholm University, 17165 Solna, Sweden; ‡ Division of Nanobiotechnology, Department of Protein Science, Science for Life Laboratory, KTH Royal Institute of Technology, 17165 Solna, Sweden; § Department of Biotechnology, Indian Institute of Technology Madras, Chennai, 600036 Tamil Nadu, India

## Abstract

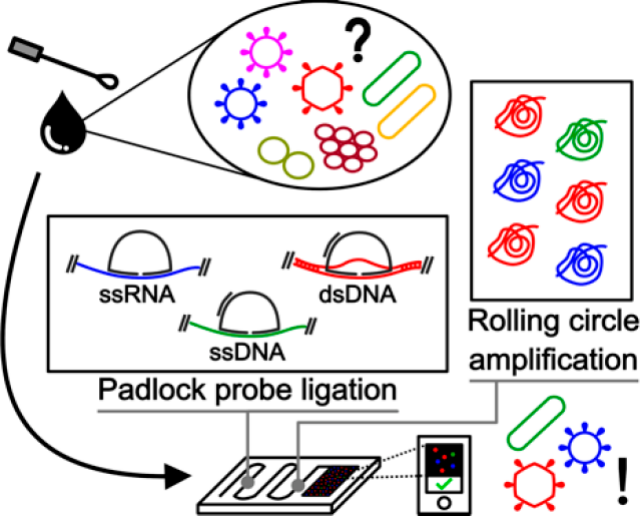

The development of robust methods allowing the precise detection of specific nucleic acid sequences is of major societal relevance, paving the way for significant advances in biotechnology and biomedical engineering. These range from a better understanding of human disease at a molecular level, allowing the discovery and development of novel biopharmaceuticals and vaccines, to the improvement of biotechnological processes providing improved food quality and safety, efficient green fuels, and smart textiles. Among these applications, the significance of pathogen diagnostics as the main focus of this Account has become particularly clear during the recent SARS-CoV-2 pandemic. In this context, while RT-PCR is the gold standard method for unambiguous detection of genetic material from pathogens, other isothermal amplification alternatives circumventing rapid heating–cooling cycles up to ∼95 °C are appealing to facilitate the translation of the assay into point-of-care (PoC) analytical platforms. Furthermore, the possibility of routinely multiplexing the detection of tens to hundreds of target sequences with single base pair specificity, currently not met by state-of-the-art methods available in clinical laboratories, would be instrumental along the path to tackle emergent viral variants and antimicrobial resistance genes. Here, we advocate that padlock probes (PLPs), first reported by Nilsson et al. in 1994, coupled with rolling circle amplification (RCA), termed here as PLP-RCA, is an underexploited technology in current arena of isothermal nucleic acid amplification tests (NAATs) providing an unprecedented degree of multiplexing, specificity, versatility, and amenability to integration in miniaturized PoC platforms. Furthermore, the intrinsically digital amplification of PLP-RCA retains spatial information and opens new avenues in the exploration of pathogenesis with spatial multiomics analysis of infected cells and tissue.

The Account starts by introducing PLP-RCA in a nutshell focusing individually on the three main assay steps, namely, (1) PLP design and ligation mechanism, (2) RCA after probe ligation, and (3) detection of the RCA products. Each subject is touched upon succinctly but with sufficient detail for the reader to appreciate some assay intricacies and degree of versatility depending on the analytical challenge at hand. After familiarizing the reader with the method, we discuss specific examples of research in our group and others using PLP-RCA for viral, bacterial, and fungal diagnostics in a variety of clinical contexts, including the genotyping of antibiotic resistance genes and viral subtyping. Then, we dissect key developments in the miniaturization and integration of PLP-RCA to minimize user input, maximize analysis throughput, and expedite the time to results, ultimately aiming at PoC applications. These developments include molecular enrichment for maximum sensitivity, spatial arrays to maximize analytical throughput, automation of liquid handling to streamline the analytical workflow in miniaturized devices, and seamless integration of signal transduction to translate RCA product titers (and ideally spatial information) into a readable output. Finally, we position PLP-RCA in the current landscape of NAATs and furnish a systematic Strengths, Weaknesses, Opportunities and Threats analysis to shine light upon unpolished edges to uncover the gem with potential for ubiquitous, precise, and unbiased pathogen diagnostics.

## Key References

- KuhnemundM.; WeiQ.; DaraiE.; WangY.; Hernandez-NeutaI.; YangZ.; TsengD.; AhlfordA.; MathotL.; SjoblomT.; OzcanA.; NilssonM.Targeted DNA sequencing and in situ mutation analysis using mobile phone microscopy. Nat. Commun.2017, 8, 139132809478410.1038/ncomms13913PMC5247573.^1^*Detection of in situ PLP-RCA using a portable smartphone microscopy platform, demonstrating potential for portability and point-of-care analysis.*
- NeumannF.; Hernandez-NeutaI.; GrabbeM.; MadaboosiN.; AlbertJ.; NilssonM.Padlock Probe Assay for Detection and Subtyping of Seasonal Influenza. Clin. Chem.2018, 64, 1704–17123025782710.1373/clinchem.2018.292979.^2^*Multiplexed Influenza detection and subtyping with PLP-RCA was demonstrated in clinical samples and benchmarked against RT-PCR.*
- SoaresR. R. G.; NeumannF.; CaneiraC. R. F.; MadaboosiN.; CiftciS.; Hernandez-NeutaI.; PintoI. F.; SantosD. R.; ChuV.; RussomA.; CondeJ. P.; NilssonM.Silica bead-based microfluidic device with integrated photodiodes for the rapid capture and detection of rolling circle amplification products in the femtomolar range. Biosens. Bioelectron.2019, 128, 68–753063407610.1016/j.bios.2018.12.004.^3^*Enrichment of PLP-RCA products in a microfluidic cartridge with integrated fluorescence signal detection allowing simple and sensitive quantification of Influenza and Ebola viruses.*
- GyllborgD.; LangsethC. M.; QianX.; ChoiE.; SalasS. M.; HilscherM. M.; LeinE. S.; NilssonM.Hybridization-based in situ sequencing (HybISS) for spatially resolved transcriptomics in human and mouse brain tissue. Nucleic Acids Res.2020, 48, e1123299074710.1093/nar/gkaa792PMC7641728.^4^*Development of a new in situ sequencing technique based on PLP-RCA allowing the multiplexed detection of more than 200 target sequences in one sample.*

## 1 Introduction

### 1.1 Isothermal Nucleic Acid Amplification

Ever since the advent of polymerase chain reaction (PCR), the means of achieving highly sensitive and precise amplification of nucleic acid sequences have paved the way toward remarkable breakthroughs in biotechnology and biomedical engineering. However, the intrinsic heating–cooling cycles up to ∼95 °C required for multiple rounds of strand denaturation, primer annealing, and polymerization limit the matrix compatibility and bioanalytical versatility of PCR. These technical challenges motivated the development of isothermal amplification techniques allowing linear or exponential amplification at a constant and preferably lower temperature. Techniques such as loop-mediated isothermal amplification (LAMP) and recombinase polymerase amplification (RPA) resort to polymerase enzymes with high strand displacement activity—avoiding the need for a denaturation or high temperature polymerization step—and/or recombinase enzymes combined with single-stranded DNA binding proteins, allowing efficient primer hybridization without temperature-driven denaturation and annealing steps.^5^ These, along with other isothermal amplification strategies,^5^ generate either short clonal amplicons with few hundreds of base pairs or nonclonal long dsDNA amplicons. Differently, rolling circle amplification (RCA), based on the mechanism of rolling circle replication found in bacteria and viruses, allows the unidirectional amplification of a circular nucleic acid template into a long single stranded molecule containing thousands of concatenated copies of the template. The efficiency and fidelity of circle replication, even with lengths down to tens of base pairs,^6^ coupled with probe circularization upon target binding, i.e., padlock probes (PLPs), can be used as a powerful nucleic acid analysis technique with single base pair specificity.^7^ PLPs, explored in detail in the subsequent section, provide the adequate boost in specificity to RCA, often lacking specificity when resorting exclusively to circle hybridization.

### 1.2 Padlock Probes: Definition, Probe Design, and Ligation Mechanism

**Figure 1:**
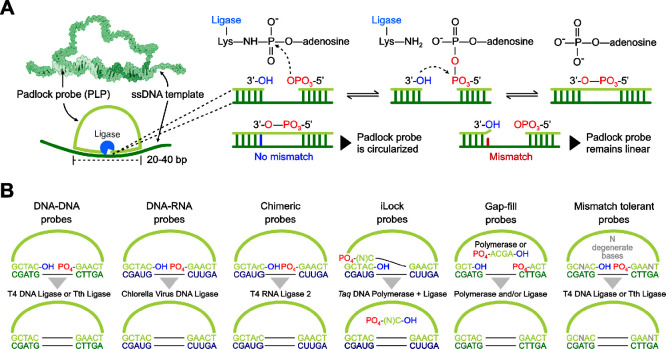
Summary of padlock probe (PLP) ligation mechanism and probe design strategies. (A) Hybridization of PLP to a single stranded DNA (ssDNA) template and ligation mechanism. The phosphorylation of the ligase prior to ligation resorts to ATP or NAD^+^ as cofactors.^11^ (B) PLP design allowing ligation to both DNA and RNA sequences. The black horizontal lines in “B” represent a standard phosphodiester bond between two bases. Molecular model in (A) was adapted with permission from ref (18). Copyright 2002 Wiley Online Library.

PLPs are linear oligonucleotides with (a) their end-sequences complementary to a target sequence and (b) a backbone with a linker sequence, hosting functionality-dependent regions such as those binding to detection or restriction oligonucleotides^7^ as discussed in detail ahead in the text. The mechanism of PLP hybridization as well as ligation is schematized in Figure 1A. A head-to-tail hybridization of the end sequences to the target with a nick in-between the end-sequences, followed by the sealing of this nick by a ligase enables target-specific probe circularization (where the PLPs become topologically linked to the target).^8^ DNA ligases mediate the joining of a 5′-phosphorylated end of a DNA strand to a 3′-end of another DNA strand upon hybridization to a complementary target DNA.^9,10^ The specificity arises from the reduced ligation efficiency in the presence of mismatches at the footprint region of the corresponding ligase, which are typically asymmetrical and extend 5–12 bp toward each end.^11^ Increased specificity is also achieved when the mismatch is near the nick site, particularly at the 3′ end for T4 and Tth ligases.^11^ Overall, toward an optimal assay performance, there are three major PLP design considerations: (1) the type of template nucleic acid, (2) the composition of the template sequence, and (3) the length of the head and tail sequences hybridizing to the target. A summary of PLP ligation strategies is schematized in Figure 1B.

The ligation of a PLP to DNA can be efficiently achieved with DNA ligases such as T4 DNA ligase or the thermostable *Tth* ligase. The latter can provide improved specificity since higher ligation temperatures can be used, maximizing hybridization stringency and mismatch discrimination. The direct detection of RNA molecules is of crucial importance in molecular diagnostics for overcoming the assay complexities and loss in yield associated with the reverse transcription.^12^ The first step toward a successful RCA-based RNA detection relies on efficient ligation during the use of PLPs for the detection of RNA sequence, for which either T4 DNA ligase or Chlorella virus DNA ligase (PBCV-1), commercially available as SplintR Ligase, have been widely used to ligate DNA oligonucleotides hybridizing to RNA strands.^13^ PBCV-1, possessing high RNA-templated DNA joining activity with enhanced fidelity and in conjunction with iLock probe/assay configurations,^14^ has been successfully used for demonstrating single nucleotide polymorphism (SNP) detection on mRNA and miRNA, with improved specificity.^14^ Likewise, RNA-templated ligation of DNA using T4 DNA ligase for accurate detection of sequence variants is also feasible, despite the need for carefully controlled reaction conditions and an inherently lower efficiency compared to PBCV-1.^15,16^ Higher catalytic efficiencies of PBCV-1 and T4 RNA ligase 2, directly reflecting on increased efficiency for RCA-based detection of targeted RNA, can be achieved by incorporating ribonucleotide substitutions in appropriate positions in the PLPs, thereby widening the scope of enzyme-assisted nucleic acid amplification tests (NAATs).^17^

### 1.3 Rolling Circle Amplification

**Figure 2:**
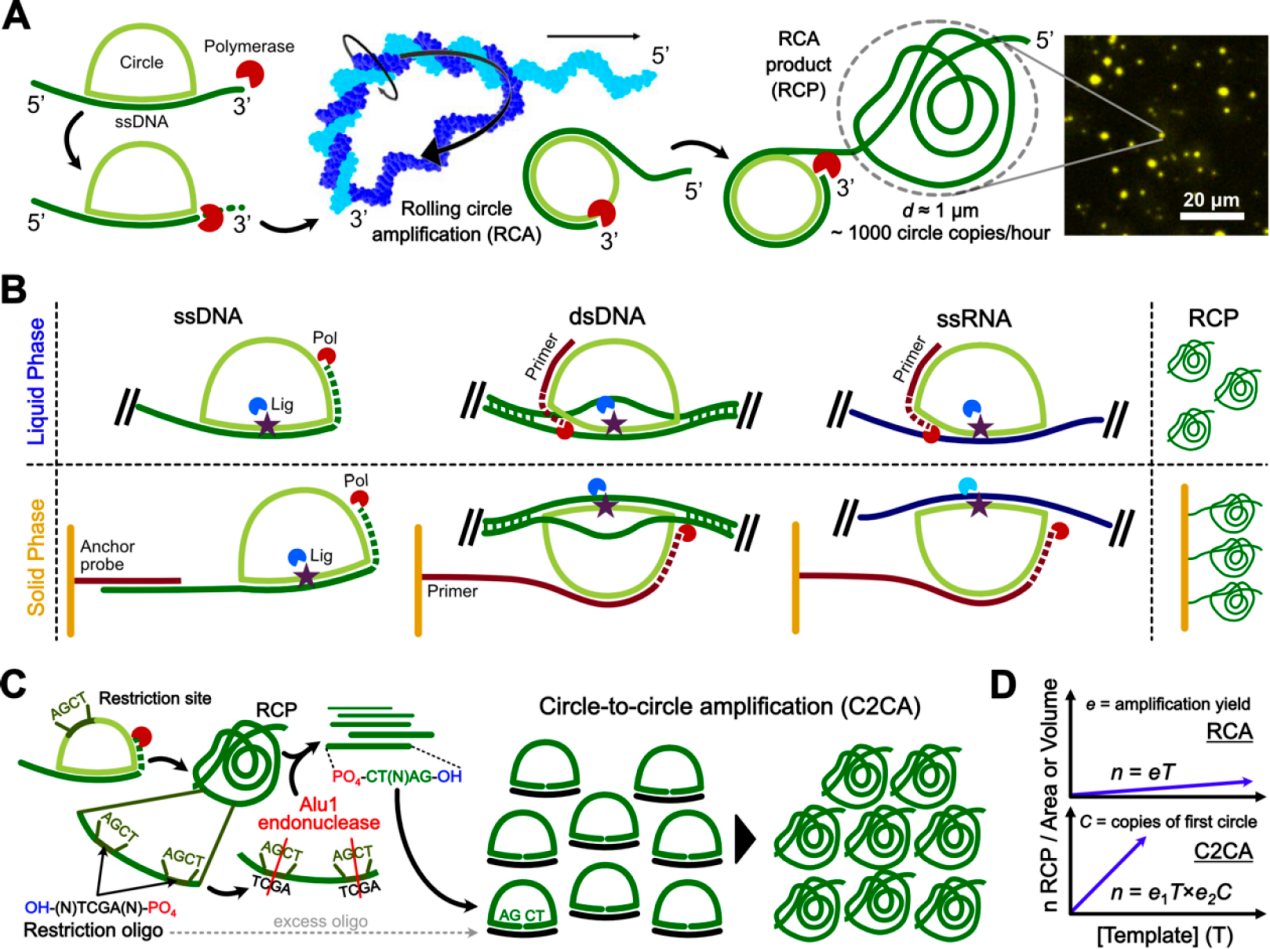
Summary of RCA mechanism and assay design strategies. (a) RCA mechanism upon PLP ligation. The polymerase molecule is larger than the circularized PLP at the scale represented in the figure. (B) RCA strategies after ligation of a PLP to ssDNA, double stranded DNA (dsDNA), or single stranded RNA (ssRNA) in solution or primed on a solid surface such as a glass slide or a microbead. (C) C2CA schematics. (D) Key variables correlating the concentration (liquid phase) or total density (solid phase) of RCPs with increasing template concentrations. The amplification yield “*e*” is a factor ranging from 0 to 1 quantifying the fraction of template-PLP pairs translated into RCPs. *C* is the total number of concatenated reverse complement circle copies in the first RCP. Molecular model in (A) was adapted with permission from ref (27). Copyright 2006 Springer Nature.

Upon ligation, PLPs circularized in a target-dependent manner can be amplified via RCA^6^ (Figure 2A). Among different polymerases, *Phi29*, a member of the B-family of replicative DNA polymerases consisting of a single 68 kDa protein, possesses the best processivity and strand displacement activity—to displace the complementary strand in double-stranded regions of a template molecule during DNA synthesis—together marking the high fidelity of the enzyme.^19^ Specifically, Phi29 can amplify a 100 bp nucleotide circular probe into a DNA concatemer with ∼1000 complementary copies of the circularized molecules in 1 h.^8^

Upon amplification, the RCA products (RCPs) collapse onto themselves owing to the polarity of DNA, when they fold into a random coil conformation. The structural collapse is attributed to the overcharging-induced DNA chain swelling, a feature prominent at a higher divalent salt concentration.^20,21^ Conditions providing an efficient collapse of RCPs result in a high spatial concentration of concatenated PLP copies, critical to provide a measurable signal in the subsequent detection step. Otherwise, RCPs which are poorly pulled together can lead to false negative or false positive signals, resulting from weak signals or multiple units of an amplicon, respectively. Inclusion of compaction oligonucleotides generates smaller products with an enhanced signal intensity and better signal-to-noise ratio.^22^ The generation of RCPs from template strands can be achieved either in solution or by having the template immobilized onto a solid phase (Figure 2B).

Whenever a higher sensitivity is required, the products from a first round of RCA can be subjected to a second RCA by including a second primer allowing restriction of the RCPs followed by circularization of the fragments, yielding a million-fold amplification of the initial template,^23^ assuming 1000 copies of the circles per round (Figure 2C). Multiple rounds can be performed in series, essentially increasing the slope of the linear amplification by a factor equal to the total number of circle copies obtained from the previous round (Figure 2D). When this is done as two or more controlled rounds of RCA, it is termed as circle-to-circle amplification (C2CA).^24^ Alternatively, the assay can be designed to generate long double-stranded products as an uncontrolled exponential reaction often producing circle-independent amplification byproducts, termed as hyperbranched RCA.^25,26^ In the latter case, the working principle is based on adding forward and reverse primers complementary to the RCP, resulting in a chain reaction of amplification catalyzed by excess *Phi29*, upstream of the circular template being amplified.

### 1.4 Detection and Assay Versatility: The Sky Is the Limit

**Figure 3:**
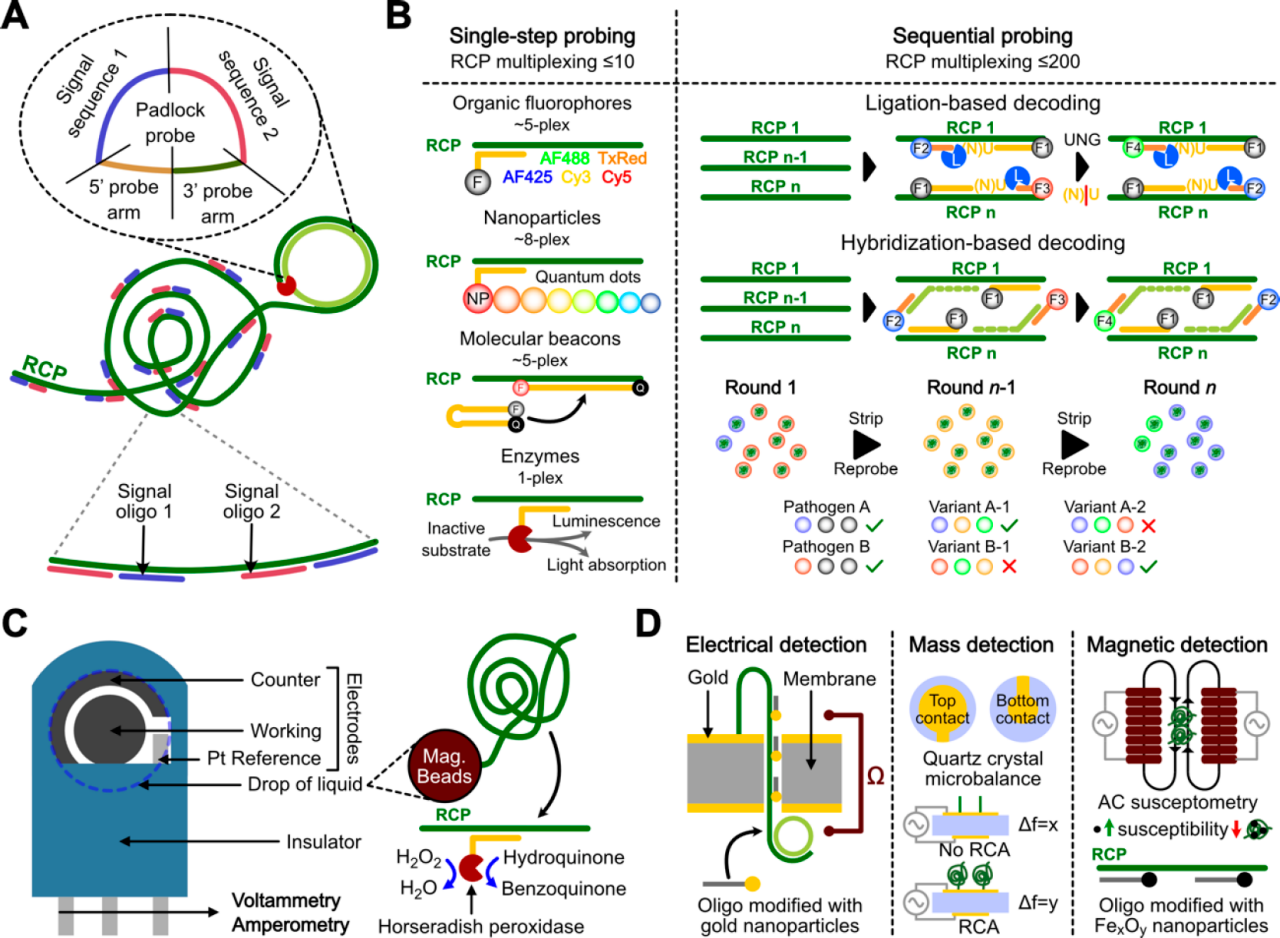
RCP labeling and detection platforms. (A) Two different labeled oligonucleotides having the same sequence as the PLP can hybridize to multiple complementary regions (signal sequences 1 and 2) in the RCP. (B) Labeling strategies for varied multiplexing requirements, i.e., number of different RCPs identified in a homogeneous mixture. Single-step probing on a single round of hybridization with labeled oligos resorting to spectrally resolved fluorescence imaging for RCP identification. Sequential probing on multiple probing and stripping cycles to decode each RCP as a series of discrete optical signals. F, fluorophore; NP, nanoparticles; L, ligase enzyme. (C) Labeling strategy for electrochemical detection. Data taken from ref (31). RCPs are generated on the surface of magnetic beads to simplify serial washing and solution exchange steps. (D) Miscellaneous physicochemical signal transduction strategies. Data taken from refs (32−34).

Since PLP-RCA amplifies the template nucleic acid into a long and repeated single-stranded oligonucleotide, self-collapsing into a spatially discrete units, its detection can be readily achieved by simply adding complementary oligonucleotides conjugated to label molecules (Figure 3A). Such complementarity can be designed in an extremely versatile manner by modifying the backbone sequence of the PLP (signal sequences 1 and 2), complementary to the RCP upon amplification. In this regard, it should be noted that the local concentration of hybridization sites provided by a single RCP (diameter of ∼1 μm) is in the order of 1 μM (assuming ∼1000 binding sites in a volume of 1 fL). Thus, such a concentration allows efficient detection with a high signal-to-noise ratio using a number of optically active labels such as organic fluorophores, even in the presence of free dye molecules in solution. This is a key feature of PLP-RCA providing an intrinsically digital detection of the target molecules upon amplification in a homogeneous solution. Besides the quantitative merits of a digital detection, i.e., high signal-to-noise ratio, calibration-free quantitative data, high sensitivity , and high precision, such a type of digital detection allows a remarkable degree of multiplexing. For example, performing a single round of RCP labeling, as much as eight different targets can in principle be detected in a single imaging step (Figure 3B) using spectrally resolvable fluorescent nanoparticles such as quantum dots. This relatively unimpressive degree of multiplexing can be dramatically expanded to more than 200 simultaneous target sequences by performing sequential probing and stripping cycles (Figure 3B). PLP-RCA, when performed on cells/tissues^28^ or, in principle, having the RCPs first immobilized on a solid phase, extends to an in situ sequencing (ISS) method^29^ with spatial resolution. In this case, multiplexing is achieved by the use of molecular barcodes in the PLP backbone, that are sequenced by either sequencing-by-ligation^28,30^ or sequencing-by-hybridization (HybISS),^4^ both reported first by our group, for precise identification of target and subsequent decoding of the expression profiles. Besides optically active labels coupled with fluorescence imaging, RCP detection is also feasible using electrochemically active enzymatic labels such as horseradish peroxidase^31^ (Figure 3C) and physicochemical transduction resorting to metal nanoparticle labels for electrical^32^ or magnetic^33^ detection as well as label-free detection using quartz crystal microbalance (QCM)^34^ (Figure 3D).

Expanding upon the versatility of PLP-RCA, by tagging antibodies with oligo sequences, all features described above can be translated to protein analysis via immunoassays, immunocytochemistry, and immunohistochemistry by the conversion of protein targets to DNA sequences, enabling sensitive protein detection. Immuno-RCA, based on the strategic implementation of PLP-RCA on antibodies, has been successfully demonstrated for sensitive detection of protein targets, including antibodies and cytokines,^35^ and also for a broader understanding of immune responses in diseases, reported for the immune-mediated thrombotic thrombocytopenic purpura.^36^ Another step ahead in DNA-based immunoassays is the proximity ligation assay,^37^ where pairs of DNA sequences are brought in proximity via their conjugated antibodies, for subsequent RCA.

## 2 Padlock Probes and Rolling Circle Amplification for Pathogen Diagnostics

Over the past decade, we have developed PLP-RCA based molecular diagnostic tools for specific, sensitive, and multiplexed pathogen detection and genotyping. A summary of key features and figures of merit of highlighted projects are listed in Table 1.

**Table 1:** Highlights of Publications from Nilsson et al. Reporting the Use of PLP-RCA for Human Pathogen Diagnostics^a^

pathogen	targets and probe set	type of amplification	readout	sensitivity	specificity	ref
fungi	10-plex PLP panel; 19 clinical samples	PLP-RCA + qPCR (in solution)	Luminex (suspension array)	median fluorescence intensity (MFI), 1000–10 000 copies per reaction	4 fungal species as specificity controls, resulted negative	Eriksson et al.^73^
bacteria and spores	1 PLP per pathogen, for 16S rDNA	PLP and C2CA for DNA and digital PLA	high performance fluorescence detector	LoD < 30 bacteria (∼qPCR) and 5 spores	demonstrated with capture probes	Göransson et al.^67^
CCHFV	1 PLP each for vRNA and for cRNA	in situ, RT, PLP-RCA	fluorescence microscopy	Combinatorial analysis (of vRNA and cRNA) yields lower RCPs	spatial specificity	Andersson et al.^42^
beta lactamase genes	3 PLPs each for 28 genes and 4 PLPs for 1 gene; 70 clinical isolates	C2CA (in solution)	microarray	10^4^ DNA copies per reaction (PCR amplicons as template); 10^7^ (25-plex)-10^9^ cells/ml	98.6% genes specificity; 88.6% BL specificity	Barišić et al.^79^
tuberculosis *rpoB*	1 wild-type and 9 mutant-specific and 1 *Mtb-*complex PLPs; 8 clinical isolates	C2CA (in solution)	volume-amplified magnetic nanobead detection assay	LoD of 10 amol with synthetic target	robust discrimination between wild type and mutant strains	Engström et al.^80^
rotavirus	58 PLPs (6 with degeneration); 22 clinical samples	RT (using gene-specific primers), C2CA (in solution)	confocal microscopy, MATLAB	LoD of 10^3^ copies with synthetic target; clinical samples of rotavirus exceed this LoD	not applicable	Mezger et al.^43^
UTI bacteria panel	1 PLP per pathogen; 88 clinical samples	C2CA (in solution)	high performance fluorescence detector (Aquila 400, Q-Linea)	100% sensitivity with accurate antibiotic susceptibility profiling	100% specificity	Mezger et al.^69^
adenovirus	1 PLP for genomic DNA, 2 PLPs for mRNAs	in situ	fluorescence microscopy	sensitivity, temporal expression profiles in relation to viral DNA content (not suitable for low copy numbers)	specificity tested 25 h post infection for viral DNA and mRNAs	Krzywkowski et al.^44^
influenza	32 PLPs; 50 clinical samples and 4 reference isolates	RT (gene-specific vs random primers) and C2CA	amplified single molecule detection (ASMD) and MRE (microfluidic RCP enrichment)	77.5% sensitivity for influenza and 73% for subtyping; LoD 18 vRNA copies	100% specificity (demonstrated by subtype-specific barcodes)	Neumann et al.^2^
HIV	5 PLPs targeting conserved regions of *gag* expressing p17 and p24 proteins. Tested with HIV isolates having different subtypes.	RT and RCA (in solution)	microfluidic affinity chromatography (RCPs on microbeads)	LoD 10–30 fM ST (0.1–0.3 amol in 10 μL)	subtype specificity; minimal nonspecific capture of labeled RCPs/oligos onto microbeads	Soares et al.^47^
Ebola	15 PLPs for single RCA clinical EBOV detection and 24 PLPs for multiplex assay; 15 clinical samples	RT and RCA (in solution)	membrane enrichment (pump vs pump-free)	vRNA + cRNA for increased sensitivity; Ct 21–24 bechmarked against RT-PCR	demonstrated with negative template, RT-negative and HeLA RNA controls)	Ciftci et al.^48^
Zika	12 PLPs (coding for *C*, *PrM*, *E*, and *NS* genes); ZIKV-infected U-87 MG cells and peripheral blood mononuclear cells	RT and C2CA (in solution)	microACE (microfluidic chromatography enrichment)	<17 copies vRNA (∼3 aM)	inherent PLP specificity	Soares et al.^49^

a ST, synthetic target; RT, reverse transcription; LoD, limit of detection.

### 2.1 Molecular Virology

We have extensively demonstrated the applications of a single round of both RCA and C2CA for the sensitive, specific, and multiplex detection of a wide range of viral pathogens.^2,38−49^ In the specific context of RNA viruses, RCA has been used for the detection of Zika Virus,^48−50^ hypervariable viruses including NDV,^46^ retroviruses such as HIV,^47,51^ hemorrhagic fever causing viruses such as CCHFV and Ebola,^48,52^ influenza,^2,53,54^ and coronaviruses such as SARS^55^ responsible for the 2003 epidemic as well as SARS-CoV-2^56−58^ causing the current pandemic. Among DNA viruses, detection of human papilloma virus (HPV),^50,59^ bacteriophages,^60^ and partial dsDNA viruses like HBV^61^ have been reported. However, despite the current status of PLP-RCA-based viral diagnostics providing a framework for sensitivity and specificity enhancement, comparable to RT-PCR as a gold standard when using C2CA,^46,49^ the compatibility of PLP-RCA enzymes with minimally processed biological samples is still largely unexplored. Likewise, for the detection of RNA viruses, efforts should be invested in achieving efficient and specific ligation of PLPs directly to the RNA template to circumvent the limitations of the reverse transcription step. Both of these directions are paramount to establish a simpler workflow comparable with PCR and other isothermal NAATs for the desired clinical impact.

### 2.2 Bacterial Detection and Genotyping of Antimicrobial Resistance

Similar to the detection of viruses, PLP-RCA has been exploited for the detection of bacteria, including their antimicrobial resistance (AMR) markers. Bacterial identification has been demonstrated for *Escherichia coli*,^62,63^*Vibrio cholerae* DNA,^64,65^*Salmonella* DNA,^66^ biowarfare agents such as spores of *Bacillus atrophaeus*(67) and for the multiplexed detection of *E. coli*, *Pseudomonas aeruginosa*, and *Proteus mirabilis* at clinically relevant concentrations.^68^ Besides bacterial identification, PLP-RCA as C2CA was demonstrated as a sensitive and specific method for antibiotic susceptibility profiling of ciprofloxacin and trimethoprim used for the treatment of uropathogenic bacteria.^69^ Furthermore, owing to the high specificity, multiplexed detection of both bacteria and AMR markers can be achieved, as demonstrated for (1) the multiplexed detection of *E. coli*, *Staphylococcus aureus*, and *P. aeruginosa*, along with two antibiotic resistance markers OXA-48 and *mecA* for carbapenem and methicillin resistance, respectively^70^ and (2) differential diagnostics of *rpoB 531* and *katG 315* mutations associated with multidrug resistant *Mycobaterium tuberculosis*.^71^ Owing to the multiplexing potential and well-established single nucleotide specificity of PLP-RCA, the profiling of AMR is a particularly interesting niche application where hundreds of genes can be simultaneously identified in a single sample, potentially paving the way for routine and widespread AMR gene testing.

### 2.3 Diagnosis of Fungal Infections

Fungal infections, conventionally dependent on morphological and physiological clinical tests demanding days to weeks, can be simplified, turned economical, and made rapid using RCA and with improved specificity and robustness,^72^ as demonstrated for the 10-plexed detection of pathogenic fungi.^73^ PLP-RCA targeting the internal transcribed spacer rDNA of *Histoplasma capsulatum*, causing a systemic fungal disease called histoplasmosis, enabled the rapid and specific detection of the fungus in clinical samples.^74^ Recently, PLP-RCA was used as an environmental screening tool for the detection of *Fonsecaea* agents causing chromoblastomycosis, a chronic cutaneous/subcutaneous mycosis with muriform cells in host tissue.^75^ Such studies provide a promising scope toward accurate and timely detection of opportunistic infections including *Candida*, *Rhizopus*, and *Mucor* (causing mucormycosis, the “black fungus” disease manifested as an aftermath incidence in the current pandemic), that infect and invade human tissues in patients with weakened immunity (as in the case of COVID-19, HIV/AIDS, other viral diseases and cancers, for example).

### 2.4 *In Situ* Pathogen Detection

The molecular detection of pathogens in a tissue context becomes crucial to understand the host–pathogen interactions and unravel infection dynamics and molecular pathogenesis, in order to devise better treatment and public health plans. Toward this goal, we have reported an in situ PLP-RCA assay for both the detection and differentiation of genomic and replicative forms of PCV2 DNA strands.^41^ PLP-RCA was also used to differentiate viral RNA and complementary RNA molecules, together with the detection of viral proteins, during different stages of the Crimean Congo Hemorrhagic Fever Virus (CCHFV) replication.^42^ We have demonstrated a RNA-labeling approach for in situ single cell analysis of Influenza A virus replication and associated co-infection dynamics with a time window in lung tissues.^45^ In another study, PLP-RCA has been used to understand viral DNA accumulation and mRNA expression profiles simultaneously in Human Adenovirus Type 5 (HAdv-5) infected cells.^44^ The extent of infection was quantifiable in both short-term lytic infections in human epithelial cells as well as long-term persistent infections in human B lymphocytes. Ultimately, it is envisioned that in situ detection using PLP-RCA can be expanded toward simultaneous highly multiplexed nucleic acid and protein analysis, providing a spatial multiomics approach spanning genomics,^76^ transcriptomics,^4^ and proteomics.^77,78^

## 3 Detection Platforms and Integration

**Figure 4:**
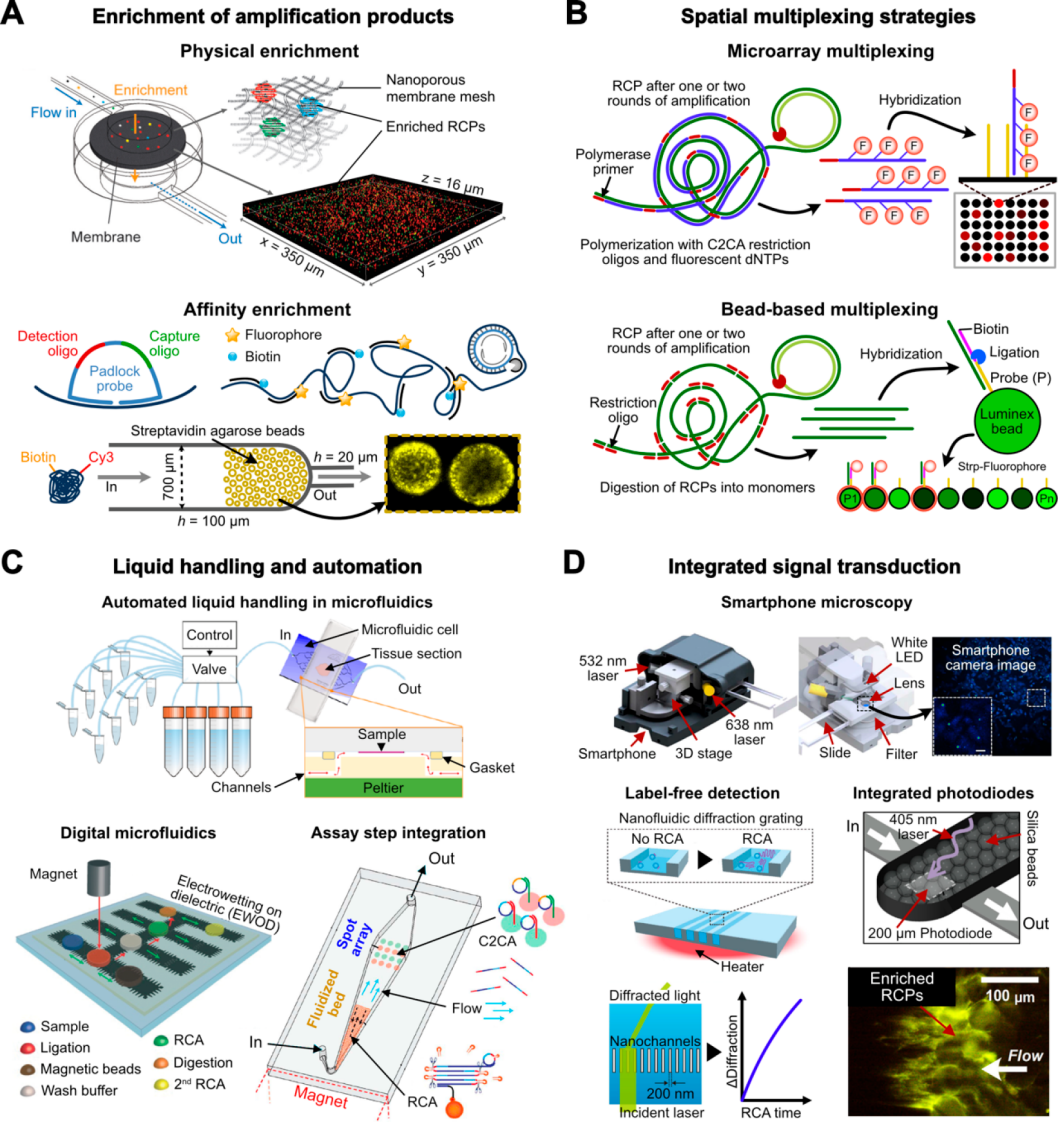
Miniaturization, integration, and automation toward point-of-care RCA-based diagnostics. (A) Enrichment of RCPs in solution based on size exclusion and/or affinity capture. These approaches allow for maximum sensitivity in digital quantification mode, i.e., counting of discrete RCPs, and expand detection into the analog mode by averaging total signal of a specific area or volume. (B) Spatially resolved multiplexing of RCA after amplification in solution. (C) Automation of liquid handling in microfluidic devices using pressure flow controllers for ISS, digitalization of RCA steps in droplets with electrowetting on dielectric (EWOD) and a magnetic fluidized bed for performing RCA. (D) Integration of optical signal transduction in microfluidic devices using smartphone microscopy, diffractometry, or miniaturized photodiodes with a total area of less than 1 mm^2^. Schematic in (A) (top) was adapted with permission from ref (70). Copyright 2017 Oxford University Press. Schematic in (A) (bottom) was adapted with permission from ref (49). Copyright 2021 Elsevier. Schematic in (C) (top) was adapted with permission from ref (83). Copyright 2019 Springer Nature. Schematic in (C) (bottom-left) was adapted with permission from ref (84). Copyright 2014 Royal Society of Chemistry. Schematic in (C) (bottom-right) was adapted with permission from ref (85). Copyright 2018 Elsevier. Schematic in (D) (top) was adapted with permission from ref (1). Copyright 2017 Springer Nature. Schematic in (D) (bottom-right) was adapted with permission from ref (86). Copyright 2016 Springer Nature. Schematic in (D) (bottom-left) was adapted with permission from ref (3). Copyright 2019 Elsevier.

Having discussed the bioanalytical merits of PLP-RCA in the context of pathogen diagnostics, a subsequent account of the current technical efforts to integrate and miniaturize the method aims at highlighting its potential value as a point-of-care (PoC) tool. Bringing up the specific case of respiratory viruses, for example, the current multiplex pathogen diagnostic toolbox used in clinical settings is dominated by multiplexed real-time quantitative PCR using integrated microfluidic cartridges from Biomérieux (BioFire FilmArray), Cepheid (GeneXpert), Luminex (NxTAG and MAGPIX), and Qiagen (QIAstat-Dx), which can typically analyze ∼20–30 target sequences per sample. Thus, the degree of multiplexing, specificity, and assay versatility provided by PLP-RCA would have a significant impact on the quality, speed, and precision of clinical decision. Furthermore, PLP-RCA would also provide a simpler alternative for the detection of AMR genes and viral variants as an alternative to expensive and labor intensive next-generation sequencing (NGS) methods. The subsequent subsections highlight advances toward tackling four key design considerations aiming at integrating PLP-RCA in miniaturized platforms (summarized in Figure 4).

### 3.1 Liquid vs Solid Phase PLP Hybridization and Enrichment of RCA Products

To face the challenge of detecting specific target sequences in a homogeneous biological matrix with adequate sample preparation, there are two different probing strategies: in solution (liquid phase) or on a solid phase (Figure 2B). Both approaches have their own unique advantages and drawbacks. Beyond more subtle differences approached in subsequent sections such as expanded multiplexing strategies and signal transduction, two fundamental differences between them concern (1) molecular availability dealing with low biomolecule concentrations in small sample volumes and (2) hybridization kinetics. Concerning the former, probing in solution implies sufficient target molecules in solution to yield measurable amplification products, while probing on a surface allows continuous perfusion of the sample from the bulk to enrich the target molecules on a confined surface area prior to amplification. However, hybridization in solution is generally 1–2 orders of magnitude more efficient than that on a solid surface^81^ due to a range of secondary interactions which are generally hard to predict and control without extensive empirical optimization. To efficiently take advantage of the improved hybridization kinetics in solution, another challenge relates to the accurate counting of 1 μm sized RCPs in large sample volumes, particularly relevant at low target concentrations. Two approaches to overcome this challenge are (1) the direct counting of individual molecules by flowing the solution through a narrow flow cell coupled with a fluorescence detector in a setup similar to flow cytometry^2^ and (2) trapping the RCPs in a small confined region resorting to physical^70^ or affinity^47^ trapping (Figure 4A). Physical trapping resorts to polycarbonate or nitrocellulose membranes with a pore size of 100 nm to filter out the RCPs in solution.^70^ On the other hand, affinity trapping works by flowing the RCPs prelabeled with a biotinylated oligo through a flow cell packed with a streptavidin-modified chromatography-grade cross-linked agarose beads.^47,49^ In either case, detection can be performed via fluorescence imaging directly on the enrichment matrix.

### 3.2 Spatial Multiplexing for Expanded Throughput

Besides the intrinsically high PLP-RCA multiplexing degree achievable with serial stripping and probing rounds, spatially resolved multiplexing can also be achieved using 2D microarrays^79^ or Luminex bead-based arrays^82^ in which each bead has a spectrally resolvable code (Figure 4B). Such an approach can be particularly useful in platforms resorting to nonoptical or enzymatic labels for RCP detection. In either of these strategies, each spot in the microarray or each bead is modified with oligonucleotide sequences complementary to a specific RCP. For the microarray, oligos used for RCP restriction are first used as primers to polymerize complementary fragments in the presence of labeled dNTPs. These labeled fragments are subsequently hybridized onto the array, and the signal intensity of each spot is converted into quantitative data. The multiplexing using Luminex beads resorts to a different strategy in which the restriction fragments of the RCPs serve as a template to ligate the complementary oligo on the beads and another complementary oligo conjugated with biotin. Essentially, the presence of the specific RCP allows the modification of the coded beads with biotin, serving as a linker for labeled streptavidin to generate the signal.

### 3.3 Liquid Handling and Miniaturization in Microfluidic Systems

Two major challenges in assay integration are, on one hand, miniaturizing the fluidic systems to economize sample and reagent usage and, on the other hand, streamlining the assay steps and liquid handling to minimize user input, thus saving labor time and reducing human error (Figure 4C). Concerning the amplification of targets in tissue samples, cells, or, in general, target molecules immobilized on solid surfaces, we have automated ISS analysis of mRNA transcripts in cells fixed on a glass slide.^83^ Specifically, the slide containing the sample is encapsulated inside a microfluidic flow cell coupled with a Peltier plate for temperature control and the sequential liquid flow is automatically operated using a pressure-flow control station and an appropriate valving system. Regarding the amplification of target sequences in solution, we have demonstrated strategies to expedite C2CA. In one example, we used a digital droplet microfluidic platform coupled with magnetic beads to automate the entire assay workflow.^84^ Furthermore, we successfully integrated both amplification steps in a single fluidic platform resorting to a magnetic fluidized bed.^85^ In this case, the first round of amplification is performed having the RCPs immobilized on magnetic beads, while the second round (after restriction) is performed in a microarray patterned on the surface of the microchannel downstream of the beads.

### 3.4 Readout Platforms and Integration of Signal Transduction

To achieve truly PoC-compatible devices, transducing RCP titers into a readable signal becomes critical (Figure 4D). For this, an epifluorescence microscope is not an ideal tool to provide a rapid response with minimal user intervention. Differently, electrochemical sensors as discussed in subection 1.4 provide a PoC-compatible signal quantification,^31,52^ albeit having limited multiplexing potential. The same applies to label-free RCP detection methods based on, for example, light diffraction patterns through a nanochannel grating,^86^ wherein the lack of specific labels requires sample splitting to achieve multiplexing. Alternatively, to take advantage of spectral multiplexing of optical methods, miniaturized photodiodes with an integrated fluorescence emission filter can be easily coupled with the fluidic system in a lensless and noncontact manner. However, due to limits in sensor miniaturization, the signal can only be acquired as an average of multiple RCPs, resulting in the loss of the spatial information and sensitivity provided by the discrete counting of RCPs.^3^ Ultimately, to realize the full potential of PLP-RCA at the PoC, a simplified microscopy apparatus resorting to a ubiquitous smartphone for image acquisition, combining both a camera and processing power, can potentially take the lead.^1^ In this regard, it becomes relevant to strike an ideal compromise between setup complexity (e.g., fluidic connections, automated liquid handling and/or imaging, minimum camera resolution, illumination sources and filter sets, lens system, etc.) and assay demands (e.g., degree of multiplexing, value of application, sensitivity requirements, etc.) to adequately meet a number of analytical challenges with PoC requirements.

## 4 Conclusions and Outlook

**Figure 5:**
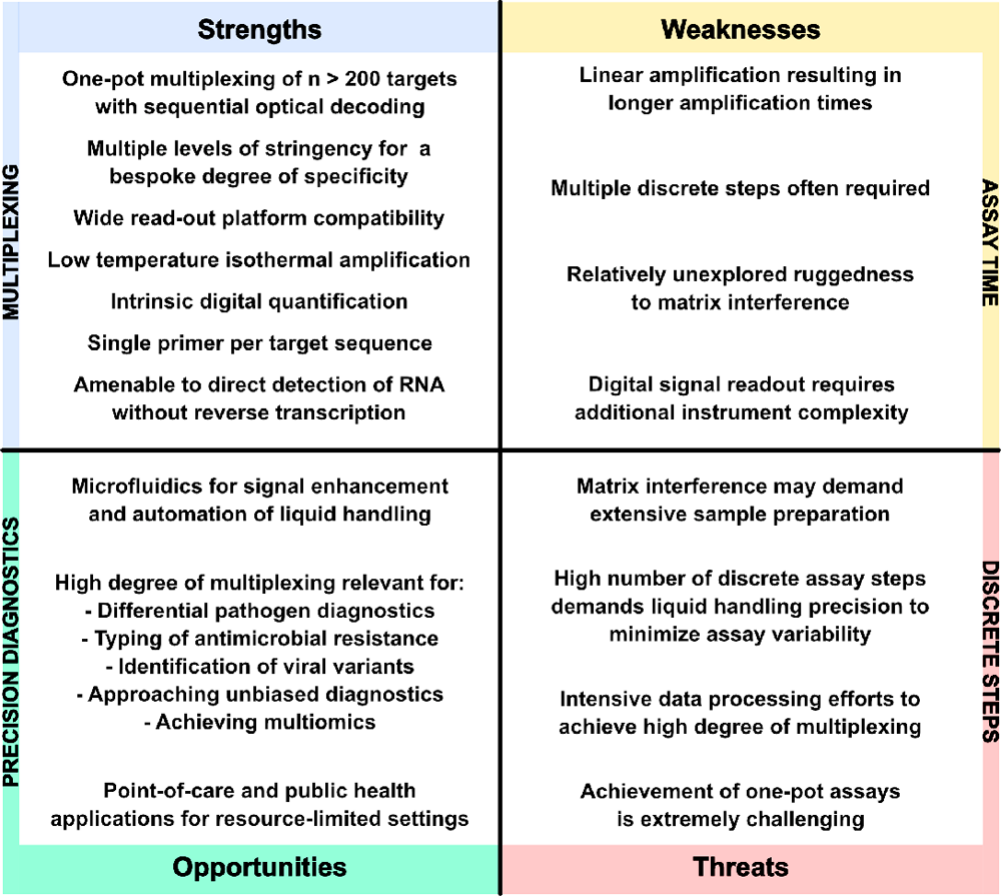
Strengths, Weaknesses, Opportunities and Threats (SWOT) of PLP-RCA for pathogen diagnostics. The term “precision diagnostics” as used herein concerns the timely and precise molecular identification of the pathogen with minimal or no bias from the symptomatic profile of the patient.

To qualitatively place PLP-RCA in the current arena of isothermal NAATs, we compare key figures of merit (Table 2) and devise a Strengths, Weaknesses, Opportunities, and Threats (SWOT) analysis to guide future directions and applications (Figure 5). The high degree of multiplexing in the range of tens to hundreds of targets combined with high specificity stands out as unique features with the potential to improve targeted diagnostics without the inherent complexity of NGS methods. The future demands for strengthening and scaling-up of PLP-RCA applications toward pathogen diagnostics become realizable via strategic streamlining of both the assay and detection platforms as well as the interfacing modules (including miniaturization and automation). Versatile sample pretreatment to avoid matrix interference, stringent hybridization and washing conditions, clinically relevant sensitivity, and direct detection of RNA (to circumvent the loss in yield and time associated with RT) are areas demanding attention from the assay perspective. The assay and detection platforms knit together with robust and efficient interfacing modules like microfluidics can effectively further the bioanalytical scope of RCA toward PoC applications including (1) detection of clinically relevant strains; (2) estimation of pathogen load during various stages of infection (including early onset); (3) pathotyping (AMR, SNPs, pathogen-associated molecular patterns, etc.); (4) differential diagnosis of broader pathogen panels; and (5) possible surveillance (supported by supporting assay/detection features), all with economy of reagents and unit test cost, relevant importantly in resource-limited settings. In this regard, we foresee, upon continued “polishing”, that PLP-RCA will be an emerging gem in the arena of NAATs providing widespread precise and unbiased pathogen diagnostics.

**Table 2:** Summary of Key Figures of Merit of PCR as the Gold Standard Amplification Method and Other Isothermal NAATs including RCA^87−89^^a^

amplification method	typical magnitude of optical multiplexing^a^	temperature	typical limit of detection	typical amplification time	clonality/type of amplicon	magnitude of amplification
PCR	<5	∼55–95 °C, ∼45 cycles	∼5 copies/reaction	∼1–1.5 h	clonal and homogeneous length, dsDNA	exponential
LAMP	1	60–70 °C, isothermal	<100 copies/reaction	30 min	nonclonal and heterogeneous length, dsDNA	exponential
NASBA	1	40 °C, isothermal	comparable to LAMP	∼1.5 h	clonal and homogeneous length, ssRNA	exponential
RPA	1	25–40 °C, isothermal	comparable to PCR	<30 min	clonal and homogeneous length, dsDNA	exponential
SDA	1	37–50 °C, isothermal	comparable to LAMP	∼1 h	clonal and homogeneous length, dsDNA	exponential
RCA	>200	25–37 °C, isothermal	∼10 copies/reaction with C2CA	1–2 h	clonal and concatenated products, ssDNA	linear

a Multiplexing capabilities using standard laboratory equipment such as real-time thermocyclers and fluorescence microscopy. Next-generation sequencing, specialized multiplexing equipment (e.g., Luminex), or strategies requiring sample splitting are not considered.
